# Biochar combined with organic and inorganic fertilizers promoted the rapeseed nutrient uptake and improved the purple soil quality

**DOI:** 10.3389/fnut.2022.997151

**Published:** 2022-09-14

**Authors:** Ming Liu, Cholidah Linna, Shumin Ma, Qun Ma, Wenfeng Song, Mingzhu Shen, Lixia Song, Kaidong Cui, Yuling Zhou, Longchang Wang

**Affiliations:** ^1^College of Agronomy and Biotechnology, Southwest University, Chongqing, China; ^2^Engineering Research Center of South Upland Agriculture, Ministry of Education, Chongqing, China

**Keywords:** biochar, fertilizer, rapeseed, soil nutrients, soil microbial community

## Abstract

Biochar is a kind of organic matter that can be added into soil to improve soil quality. To study the effect of biochar combined with organic and inorganic fertilizers on rapeseed growth and purple soil fertility and microbial community, a completely randomized block design was designed with three levels of biochar (B0: no biochar, B1: low-rate biochar, B2: high-rate biochar); two levels of inorganic fertilizers (F1: low-rate inorganic fertilizer; F2: high-rate inorganic fertilizer); and two levels of organic fertilizers (M1: no organic fertilizer; M2: with organic fertilizer). All combinations were repeated three times. The combined application of biochar and organic and inorganic fertilizers could improve soil pH, soil fertility and soil microbial community richness: The pH of B1F2M1 increased 0.41 compared with the control, the nitrogen, phosphorus and potassium content increased by 103.95, 117.88, and 99.05%. Meanwhile, soil microbial community richness was also improved. Our research showed that biochar could promote the Nutrient Uptake of rapeseed, and the combined application of biochar with organic and inorganic fertilizers could improve soil fertility and increase microbial diversity. Low-rate biochar combined with organic fertilizer and low-rate inorganic fertilizer was the most suitable application mode in rapeseed production in purple soil area of Southwest China.

## Introduction

Agriculture production faces the challenge to feed 9.8 billion humans in 2050 as well as climate change (1). The healthy soil is attentively correlated with agricultural production. Management of soil quality is critical to maintaining agro-ecosystem services (2). The challenges of the agriculture sector to escalating production are natural resources degradation, small landholder, and the effect of climate change (3).

In order to increase yield, the application of inorganic fertilizer is the simplest and most effective means. Cui et al. (4) reported that from 1961 to 2011, China's agricultural production had been strengthened by the Green Revolution with cereal production escalating by 3.9 times. Increased yield in the Green Revolution was associated with the high application of chemical fertilizers, pesticides, and irrigations as well as with the critical problems of soil degradation, water contamination and air pollution (5, 6). Overuses of inorganic fertilizers, particularly P and N that mobilized and flowed into groundwater through runoff and leaching, have determined water eutrophication and soil acidification (7). In the face of increasing food demand, there is a need to change the concept of maintaining high productivity while reawakening the protection of natural resources such as soil, water and air (5). Sustainable soil management is a must for control and development of modern agriculture (8).

As a substitute for inorganic fertilizers, organic fertilizers had a good ecological protection effect (9). Liu et al. (10) showed that long-term use of organic fertilizers could increase the content of soil organic matter and significantly increased soil microbial biomass. Organic fertilizers was rich in nutrients and met the requirements of ecological agriculture development. Studies showed that organic fertilizers contained a large amount of carbohydrates, which provided a sufficient carbon source for the growth of soil microorganisms (10). However, organic fertilizers also had some disadvantages such as heavy metal residues and slow onset of effects.

Biochar, a high-carbon material, is charred by biomass such as wood, grass, manure, and agricultural wastes through pyrolysis process (11, 12). Due to the recalcitrance of its chemical structure, biochar provides more stable soil C and stays in the soil for a longer time (13–15). Moreover, biochar not only improved soil properties but also mitigated climate change with soil carbon sequestration (7, 16). Lehmann et al. (11) described that the stability of biochar could escalate nutrient availability beyond a fertilization effect. Biochar has been widely proposed as a strategy to improve soil quality, to increase crop productivity and to address climate change and soil degradation (17–21).

At present, many studies of biochar have already been done by researchers. Study by Van Zwieten et al. (17) revealed that biochar has improved soil quality and crop growth. Meanwhile, Shamim et al. (22) showed that application of biochar together with full rate of NPK fertilizer in alkaline soil increased biomass and seed yield of rapeseed by 391 and 377%, respectively compared with no biochar or inorganic fertilizer. Bruun et al. (1) has proved that biochar significantly increased the density of roots in the 40–80 cm depth interval in barley. Biochar amendment also increased the root biomass and developed root system extensively in sandy and sandy loam soil (23, 24). Recently, Qian et al. (25) demonstrated that elongation of the wheat roots was promoted by adding 5% biochar processed from rice straw by pyrolysis at 400°C. Especially in recent years, the researches of biochar in soil improvement, especially in the soil environment of farmland and fruit trees, had become more and more extensive (26). Mousavi et al. (27) showed that biochar was the most promising option for addressing environmental problems such as soil degradation and food production and highlighted the response of biochar in the soil-plant-environment continuum.

Rapeseed is an important edible oil crop in China. The rapeseed planting area is 1.35 billion hectares, and the output is about 4.5–5 million tons, but the supply of rapeseed oil is insufficient (28). Inorganic fertilizers, especially nitrogen fertilizers, had controlled rapeseed production (29). Excessive use of chemical fertilizers had affected the environment, human health and increased production costs (30). Improved management practices were needed to reduce the application of inorganic fertilizers. Biochar acts as a sorbent for organic and inorganic fertilizers, which could increase crop yields and reduce fertilizer use (31). Although many field experiments and greenhouse experiments had confirmed that biochar improves crop yield, there was a lack of research on the effects of biochar combined with inorganic and organic fertilizers on the growth and development of rapeseed in purple soil. Purple soil is a special soil type in China. It is the primary soil on the purple-red sandstone and shale rich in calcium carbonate in the subtropical region. The purpose of this study was: (1) to evaluate the effects of biochar application on the growth of rapeseed in purple soil areas, and (2) to investigate the effects of combined application of biochar and inorganic and organic fertilizers on soil fertility and microbial communities in purple soil.

## Materials and methods

### Experimental area

The experiment was conducted using pot experiment in the green house of College of Agronomy and Biotechnology (CAB), Southwest University (SWU), China, during September 2016 to May 2017. There was no additional supplementary light in the greenhouse, and the light intensity and photoperiod in the greenhouse were consistent with the external environment. The research area was located at 220 m in altitude, 29°49′32″N in latitude, and 106°26′02″E in longitude. Measurement of soil, biochar and plant parameters was carried out in Key Laboratory of Crop Quality Improvement of Chongqing Municipality, CAB, SWU, China. The main physical and chemical properties of the soil are: soil bulk density 1.21 g·cm^−3^, pH value 6.47, organic matter content 28.00 g·kg^−1^, total nitrogen content 1.68 g·kg^−1^, total phosphorus content 1.46 g·kg^−1^, the total potassium content is 34.54 g·kg^−1^, the available phosphorus is 18.13 mg·kg^−1^, the available potassium is 270.23 mg·kg^−1^, and the alkaline hydrolyzable nitrogen is 35.23 mg·kg^−1^.

### Experimental design

A pot experiment was conducted in Randomized Complete Block Design (RCBD) with 3 × 2 × 2 treatments, with three replications. The first treatment was rate of biochar(B): B0, B1, and B2. The second treatment was fertilizer (F): F1, and F2. The third treatment was organic fertilizer (M): M0 and M1. Table 1 showed the twelve treatment combinations. Table 2 showed the detail information of biochar, soil, inorganic fertilizer and organic fertilizer in each pot.

**Table 1:** Treatment combinations of biochar, inorganic fertilizer, and organic fertilizer.

**Treatment**	**Inorganic fertilizer** **(30 N, 87.5 P**_**2**_**O**_**5**_**, 60 K**_**2**_**O)** **(kg/ha) (F1)**		**Inorganic fertilizer** **(60 N, 175 P**_**2**_**O**_**5**_**,120 K**_**2**_**O)** **(kg/ha) (F2)**	
	**Organic fertilizer 0 t/ha (M0)**	**Organic fertilizer 4.5 t/ha (M1)**	**Organic fertilizer 0 t/ha (M0)**	**Organic fertilizer 4.5 t/ha (M1)**
Biochar 0 t/ha(B0)	B0F1M0	B0F1M1	B0F2M0	B0F2M1
Biochar 35 t/ha(B1)	B1F1M0	B1F1M1	B1F2M0	B1F2M1
Biochar 50 t/ha(B2)	B2F1M0	B2F1M1	B2F2M0	B2F2M1

**Table 2:** Treatment combinations of biochar, inorganic fertilizer, and organic fertilizer in each pot.

**Treatment**	**Urea (g)**	**P_2_O_5_ (g)**	**K_2_O(g)**	**Organic fertilizers (g)**	**Biochar (g)**	**Soil (g)**	**Total (g)**
B0F1M0	0.1	0.3	0.2	0	0	4999.4	5,000
B0F1M1	0.1	0.3	0.2	15	0	4984.4	5,000
B0F2M0	0.2	0.6	0.4	0	0	4998.8	5,000
B0F2M1	0.2	0.6	0.4	15	0	4983.8	5,000
B1F1M0	0.1	0.3	0.2	0	116	4883.4	5,000
B1F1M1	0.1	0.3	0.2	15	116	4868.4	5,000
B1F2M0	0.2	0.6	0.4	0	116	4882.8	5,000
B1F2M1	0.2	0.6	0.4	15	116	4867.8	5,000
B2F1M0	0.1	0.3	0.2	0	166	4833.4	5,000
B2F1M1	0.1	0.3	0.2	15	166	4818.4	5,000
B2F2M0	0.2	0.6	0.4	0	166	4832.8	5,000
B2F2M1	0.2	0.6	0.4	15	166	4817.8	5,000

Total weight of soil, biochar, and organic fertilizer in each pot was 5,000 g. They were mixed with inorganic fertilizer as treatment before transplanting. The seeds of rapeseed were provided by Rapeseed Research Institute, CAB, SWU, China. According to local planting traditions: rapeseed was sown in September 2016, and the rapeseed variety was “Zhongshuang No. 11”. Each pot was transplanted with 1 plant. The plants were watered once every 5 days according to the daily temperature. The amount of water added in each pot was the same, and the amount of water added in each pot was 200–600 ml, to made sure the relative soil moisture content was controlled at about 60% of the saturated moisture. The saturated water content of soil was measured before planting, and the weight of 60% of the saturated water content with the pot was calculated. Weighed the pot when watering, subtract the weight of the plant and make up to 60% of the saturated water content with water. The temperature of glasshouse ranged from 20 to 25°C and relative humidity was 50 to 90% during the entire growth period.

Biochar (carbonized corncob) was obtained commercially from Nanjing Qinfeng Straw Technology Co., LTD., China. Based on the information from the manufacturer, corncob was prepared at a pyrolysis temperature of 400°C. Inorganic fertilizers used were urea, P_2_O_5_, and K_2_O as sources of mineral nitrogen, phosphate, and potassium, respectively. The organic fertilizer used in the experiment was the ZhenGeng biological organic fertilizer provided by Beijing Xingpeng Agricultural Development Co., Ltd. The main component of organic fertilizer was pig manure, and the main technical parameters were: organic matter content was >50%, Amino acid + nucleotide was >3%, moisture was lower than 18.0%, effective viable count was >50 million/g, Zn + B + Fe + Mn + Mo + Si was >0.1%. The tested purple soil was collected from the Experiment Farm of SWU, China. The soil was air-dried and sieved to pass through 2 mm. The plastic pots used in the experiment were 23 cm in diameter and 22 cm tall with a total soil volume of 5,000 gram. The basic physical and chemical properties of biochar were: pH was 9.90 ± 0.04, EC was 0.94 ± 0.01 ms·cm^−1^, total nitrogen was 5.76 ± 0.23 g·kg^−1^, total phosphorus was 3.75 g·kg^−1^, total potassium was 21.15 ± 0.17 g·kg^−1^, CEC was 34.49 ± 5.16 cmol·kg^−1^, and organic carbon was 439.01 ± 40.06 g·kg^−1^.

### Plant and soil analysis

The data of rapeseed plants were collected at four stages of growth: vegetative stage, bud stage, flowering stage, and maturity stage. Plant dry weight was measured gravimetrically with an electronic scale.

Rapeseed plants were harvested at the end of May 2017. The plants were removed from the pot and divided into stems and pods while roots were washed thoroughly. Dry matter of plant samples was obtained after being dried in an oven at 75°C for 48 h.

Kjeldahl method was used for determination of total nitrogen content in plant tissues. Phosphorus content was determined by vanadium molybdenum yellow absorbance method. Potassium content was determined by flame photometer method (32).

Soil samples were collected at the same stages as plant samples. They were air-dried and then sieved to pass through 2 mm. Soil pH (H_2_O) was measured in 1:5 soil to water ratio with pH digital apparatus. Kjeldahl method was used for determination of soil total nitrogen content. Total phosphorus was determined using molybdenum antimony anticolorism method. Soil total potassium was determined using flame photometer method (33, 34). The ready kits provided by Nanjing Jiancheng Bioengineering Institute, China was used for determining soil organic matter (SOM). The test of SOM was carried out following the procedure mentioned with the detection kits. The 1 M NH_4_OAc saturation method at pH7 was used to measure cation exchange capacity (CEC) (35).

The soil samples for soil microbial community were restored in −80°C fridge to keep fresh before test. Phospholipid fatty acids (PLFA) method was used for measurement of soil microbial diversity. Phospholipid fatty acids were extracted from fresh sieved soil at the maturity stage. Procedure standard method of PLFA followed the literature described by Zalles and Bai (36), Zelles (37), Ringelberg et al. (38), and Buyer and Sasser (39). The recognized PLFAs were appointed into a microbial group, such as eukaryotes, Poly-unsaturated fatty acids, gram-positive bacteria (branched saturated fatty acids), gram-negative bacteria (monounsaturated fatty acids) and protozoa labeled as 20:3 and 20:4.

### Statistical analysis

The factorial ANOVA technique was used to analyze the data. Only the significant ANOVA results were fixed with Least Significant Different (LSD) test with probability of 0.05, which was performed by SPSS 17.0 (SPSS Inc., Chicago, IL, USA). Graphics of the data was made using Microsoft Excel. Redundancy analysis (RDA) was conducted using CANOCO 5.0.

## Results

### The effects of biochar combined with organic and inorganic fertilizers on rapeseed nutrients and oleic acid content

**Figure 1:**
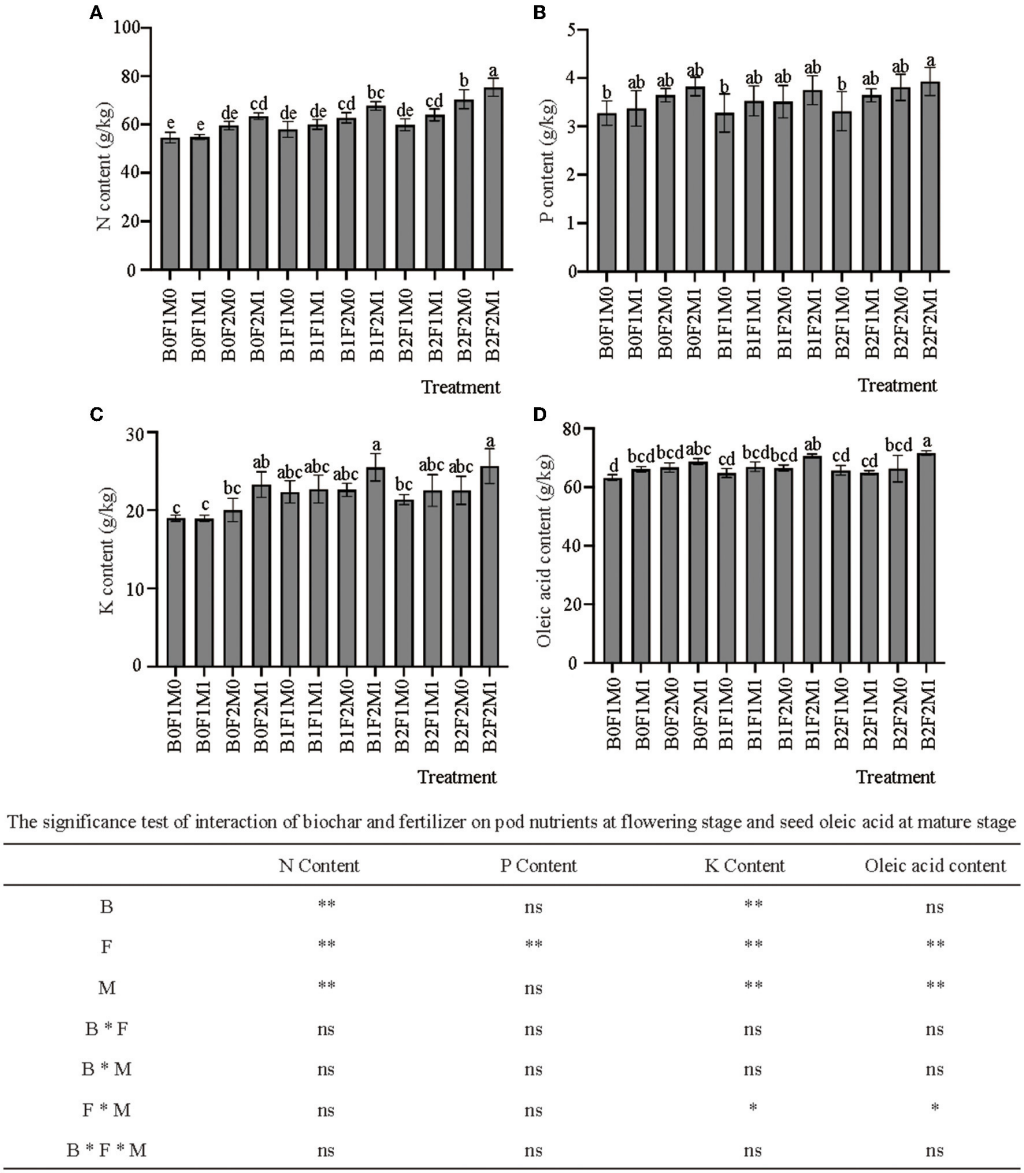
Effects of biochar, inorganic fertilizer and organic fertilizer on pod nutrition at flowering stage **(A–C)** and seed oleic acid content at mature stage **(D)**. Bars followed by the same letter are not significantly different according to LSD-test (*P* ≤ 0.05). Bars represent standard errors of the means. * and ** Significant difference at *p* = 0.05 and *p* = 0.01, respectively, and ns indicates no significant difference.

The application of biochar, inorganic and organic fertilizers had different effects on various nutrients and oleic acid in pods (Figure 1). At the flowering stage, the contents of nitrogen, phosphorus and potassium in the pods of B2F2M1 were the highest, being 38.22, 20.18, and 35.14% higher than those of B0F1M0, respectively. Under the same amount of biochar application, the nitrogen, phosphorus and potassium contents in the pods under different fertilizer treatments were: organic and inorganic fertilizers > inorganic fertilizer > no fertilizer. The nitrogen content of the pods of B0F2M1 increased by 15.83% compared to B0F1M1 and 6.57% compared to B0F2M0; the nitrogen content of pods of B1F2M1 increased by 12.66% compared to B1F1M1 and 7.91% compared to B1F2M0; and the nitrogen content of pods of B2F2M1 increased by 17.89% compared to B2F1M1 and 7.07% compared to B2F2M0 (Figure 1A). The phosphorus content of pods of B0F2M1 increased by 13.33% compared to B0F1M1 and 4.75% compared to B0F2M0; the phosphorus content of pods of B1F2M1 increased by 6.33% compared to B1F1M1 and 6.93% compared to B1F2M0; and the phosphorus content of pods of B2F2M1 increased by 7.68% compared to B2F1M1 and 3.06% compared to B2F2M0 (Figure 1B). The potassium content of pods of B0F2M1 increased by 23.14% compared to B0F1M1 and 16.34% compared to B0F2M0; the potassium content of pods of B1F2M1 increased by 12.30% compared to B1F1M1 and 12.73% compared to B1F2M0; and the potassium content of pods of B2F2M1 increased by 13.87% compared to B2F1M1 and 13.82% compared to B2F2M0 (Figure 1C).

This study showed that the application of biochar combined with organic and inorganic fertilizers increased the oleic acid content of rapeseed at maturity (Figure 1D), and the oleic acid content of B2F2M1 was significantly higher than B2F1M1, being increased by 10.24 and 7.86% compared to B2F2M0.

### Effects of biochar combined with organic and inorganic fertilizers on soil fertility

**Figure 2:**
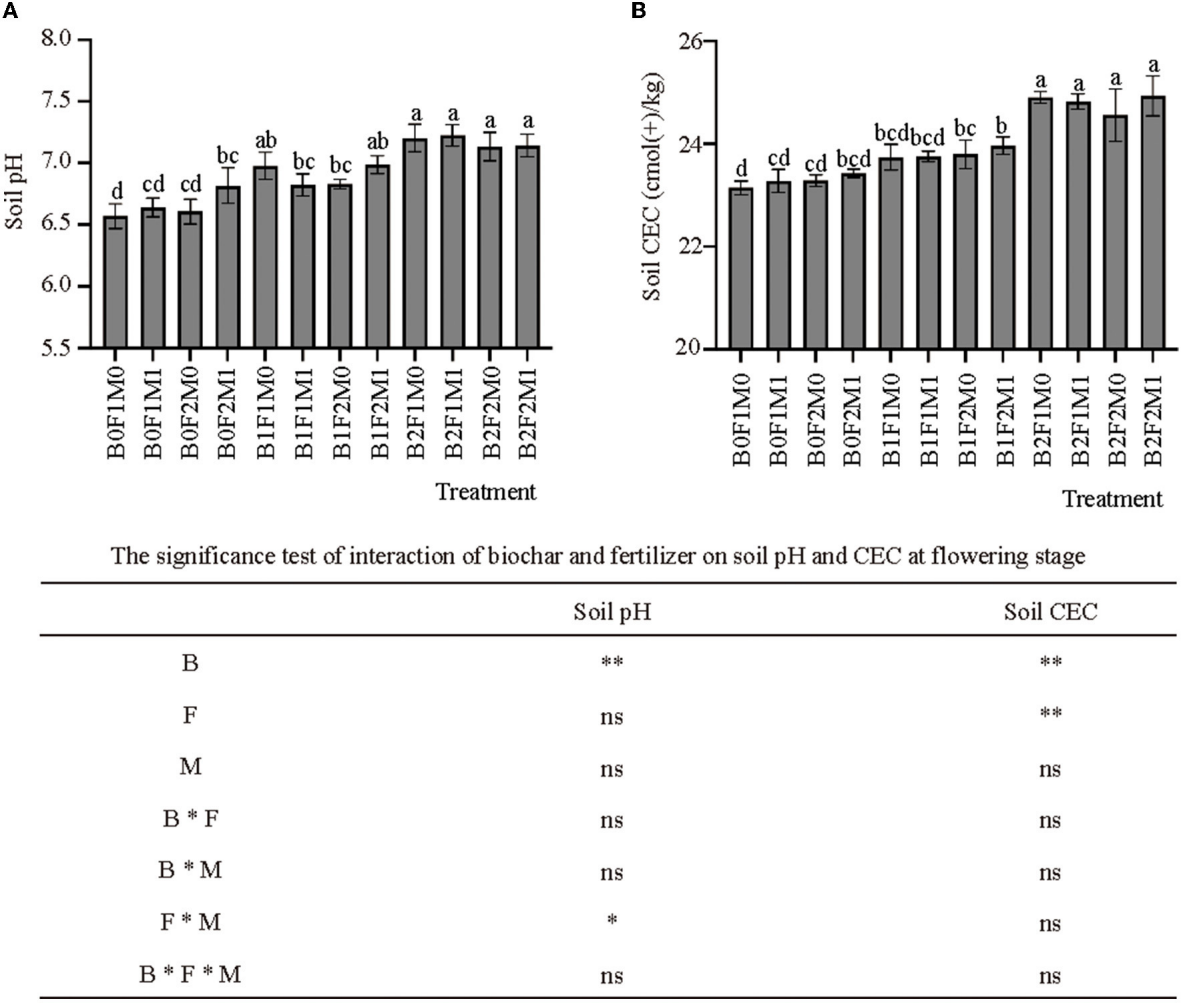
Effects of biochar, inorganic fertilizer and organic fertilizer on soil pH **(A)** and CEC **(B)** at flowering stage. Bars followed by the same letter are not significantly different according to LSD-test (*P* ≤ 0.05). Bars represent standard errors of the means. * and ** Significant difference at *p* = 0.05 and *p* = 0.01, respectively, and ns indicates no significant difference.

The application of biochar, inorganic and organic fertilizers influenced soil pH during flowering stage (Figure 2A). The application of biochar significantly increased the soil pH. The soil pH of the high-rate application of biochar was significantly higher than that of the other treatments, and the soil pH of B2F2M1 was 4.79% higher than that of B0F2M1. This study showed that the application of organic fertilizer could raise the pH of soil. The pH of B0F2M1 was 0.16 higher than that of B0F2M0, and the pH of B1F2M1 was also 0.16 higher than that of B1F2M0. Meanwhile, the application of biochar significantly increased the CEC of the soil (Figure 2B). The soil CEC of B1 and B2 was 2.27 and 6.53% higher than B0, respectively.

**Figure 3:**
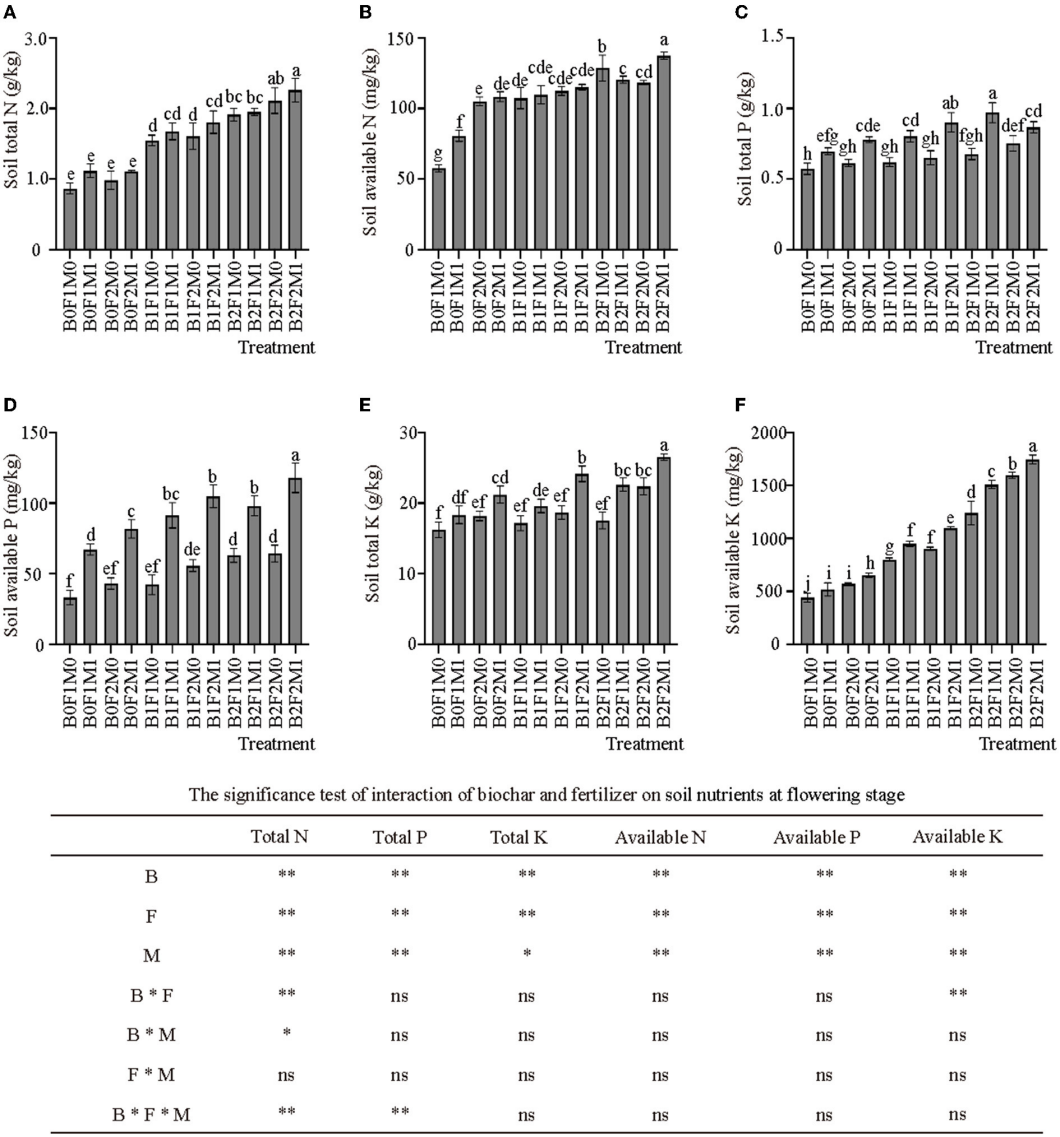
Effects of biochar, inorganic fertilizer and organic fertilizer on soil nutrients at flowering stage **(A–F)**. Bars followed by the same letter are not significantly different according to LSD-test (*P* ≤ 0.05). Bars represent standard errors of the means. * and ** Significant difference at *p* = 0.05 and *p* = 0.01, respectively, and ns indicates no significant difference.

The application of biochar with inorganic and organic fertilizers significantly affected soil nitrogen content at flowering stage (Figures 3A,B). The application of biochar significantly increased the total nitrogen content in the soil. The total nitrogen in the soil treated with high rate of biochar and low rate of biochar were 101.96 and 62.71% higher than that without biochar treatment, respectively. Meanwhile, the total nitrogen content of B2F2M1 was the highest, being 160.97% higher than B0F1M0. The application of biochar combined with organic and inorganic fertilizers had a great effect on soil available nitrogen. Compared with B0F1M0, the soil available nitrogen of B0F1M1 and B0F2M0 increased by 37.93 and 81.09% respectively, and B1F1M1 increased by 75.86% compared with B0F1M0.

The application of organic fertilizer had a significant effect on the content of soil total phosphorus and soil available phosphorus (Figures 3C,D). The total phosphorus content of the soil treated with organic fertilizer was significantly higher than that of other treatments. The soil total phosphorus content of B0F1M1 was 21.51% higher than B0F1M0. B1F1M1 was 29.03% higher than B1F1M0; and B2F1M1 was 43.39% higher than B2F1M0. Similar to soil total phosphorus, the soil available phosphorus content in the treatment with organic fertilizer was significantly higher than that in the other treatments. The soil available phosphorus content of B0F1M1 was 103.03% higher than B0F1M0. B1F1M1 was 115.75% higher than B1F1M0; and B2F1M1 was 55.56% higher than B2F1M0.

The application of biochar and inorganic and organic fertilizers significantly affected soil potassium content at flowering stage (Figures 3E,F). The total soil potassium content in B0F2M1, B1F2M1, and B2F2M1 were 30.59, 48.64, and 63.24% higher than B0F1M0, respectively. Meanwhile, different biochar application rates also had effect on soil total potassium content, and the soil total potassium content of B1 and B2 was 7.57 and 19.33% higher than B0, respectively. The effect of biochar on soil available potassium content was different from that of biochar on soil total potassium content: soil available potassium exhibited significant differences under different biochar application. The soil available potassium content of B1 and B2 were 71.81 and 179.28% higher than B0, respectively. The application of biochar greatly increased the available potassium content in the soil.

**Figure 4:**
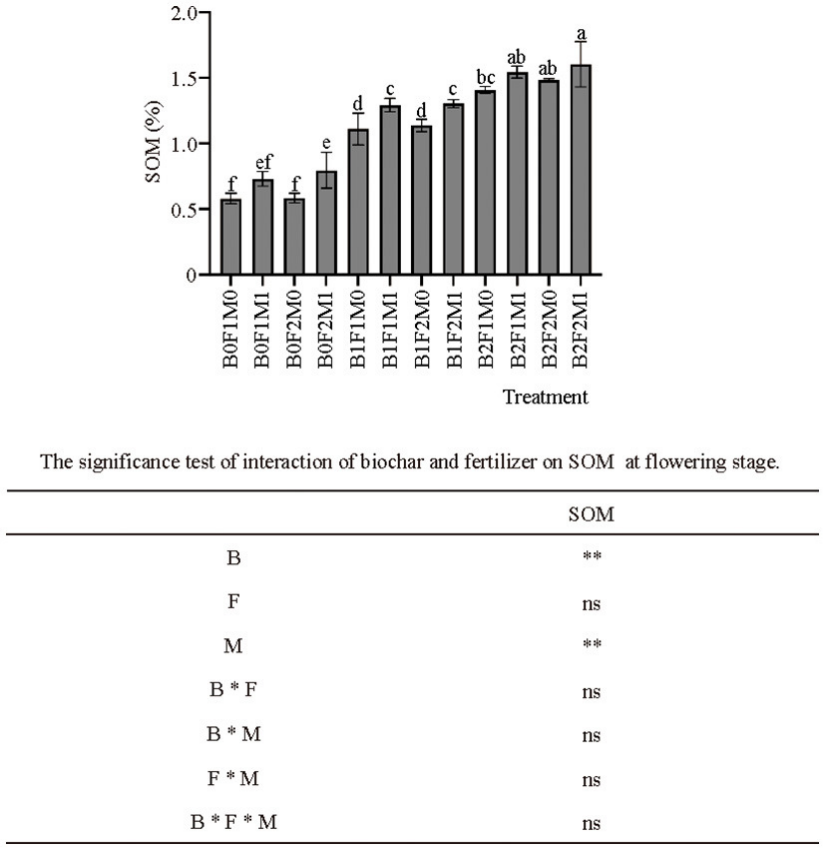
Effects of biochar, inorganic fertilizer and organic fertilizer on SOM at flowering stage. Bars followed by the same letter are not significantly different according to LSD-test (*P* ≤ 0.05). Bars represent standard errors of the means. ** Significant difference at *p* = 0.01 and ns indicates no significant difference.

The application of biochar with inorganic and organic fertilizers significantly affected the content of SOM (Figure 4). The application of biochar significantly affected SOM: the SOM of B1 and B2 were 80.17 and 124.55% higher than B0, respectively. At the same time, the application of organic fertilizer also significantly increased the SOM: the SOM of B0F1M1 was 25.86% higher than B0F1M0; B1F1M1 was 16.21% higher than B1F1M0; and B2F1M1 was 9.72% higher than B2F1M0.

### The effects of biochar combined with organic and inorganic fertilizers on soil microbial community

**Figure 5:**
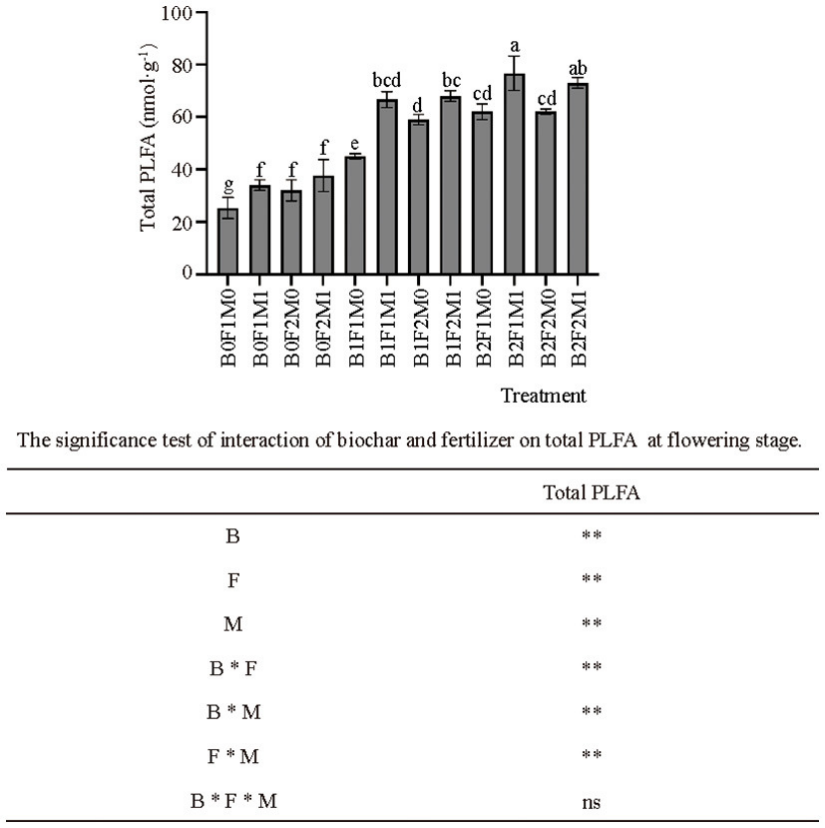
Effects of biochar, inorganic fertilizer and organic fertilizer on soil PLFA at flowering stage. Bars followed by the same letter are not significantly different according to LSD-test (*P* ≤ 0.05). Bars represent standard errors of the means. ** Significant difference at *p* = 0.01 and ns indicates no significant difference.

The application of biochar with inorganic and organic fertilizers significantly affected the soil PLFA (Figure 5). First, the application of biochar significantly increased the soil PLFA: the soil PLFA of B1 and B2 was 84.99 and 112.16% higher than that of B0, respectively. At the same time, the application of organic fertilizers also significantly increased the total soil PLFA: the soil PLFA of B0F1M1 was 34.22% higher than B0F1M0; B1F1M1 was 48.15% higher than B1F1M0; and B2F1M1 was 23.64% higher than B2F1M0.

**Figure 6:**
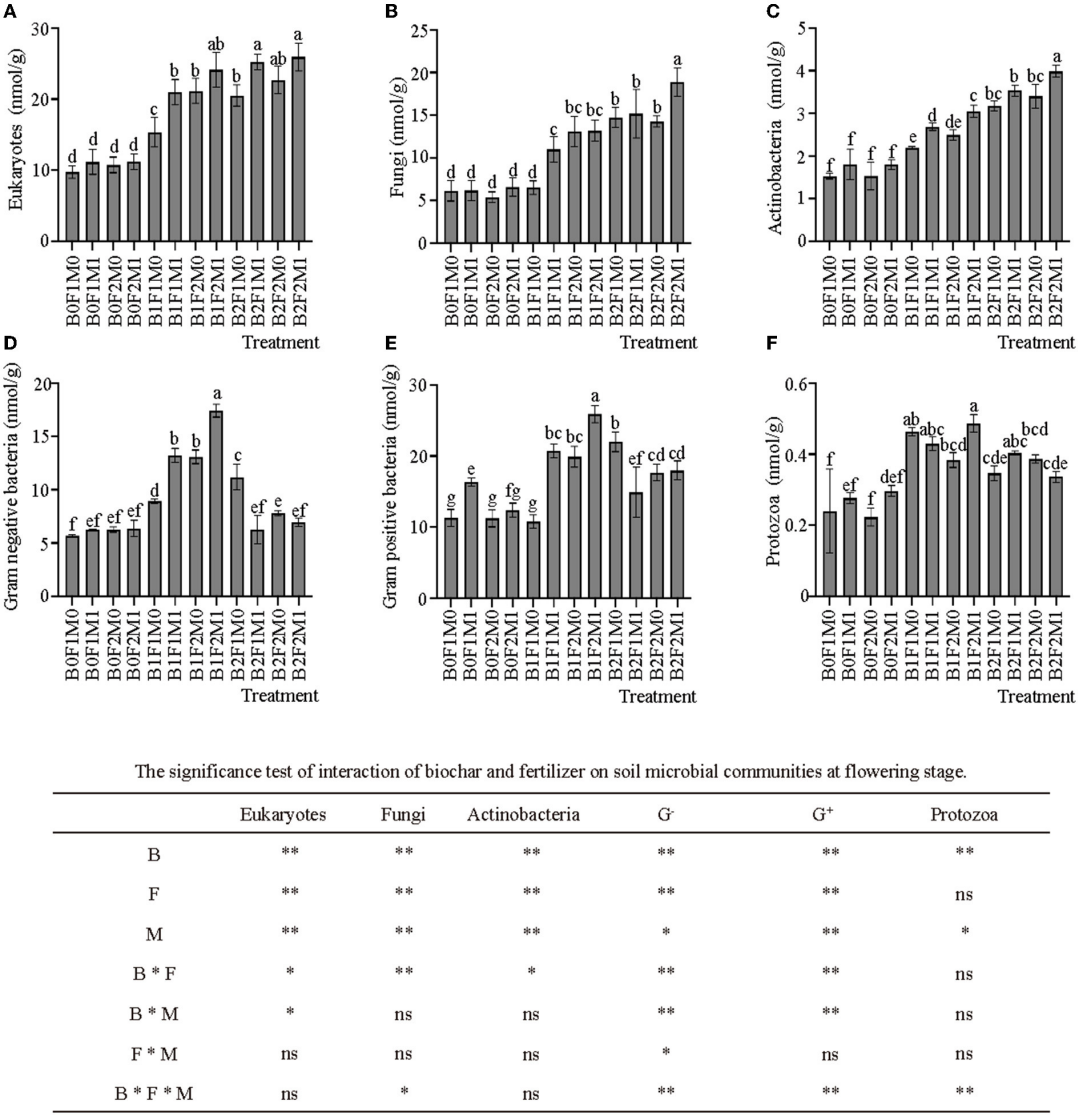
Effects of biochar, inorganic fertilizer and organic fertilizer on soil microbial communities at flowering stage **(A–F)**. Bars followed by the same letter are not significantly different according to LSD-test (*P* ≤ 0.05). Bars represent standard errors of the means. * and ** Significant difference at *p* = 0.05 and *p* = 0.01, respectively, and ns indicates no significant difference.

The application of biochar with inorganic and organic fertilizers significantly affected soil microbial community composition (Figure 6). The application of biochar significantly increased the number of eukaryotes (Figure 6A): the eukaryotes of B1 and B2 were 90.51 and 120.30% higher than B0, respectively. At the same time, the application of organic fertilizer also increased the number of eukaryotes: the eukaryotes of B0F1M1 was 14.72% higher than B0F1M0; B1F1M1 was 36.74% higher than B1F1M0; and B2F1M1 was 23.08% higher than B2F1M0. The application of biochar significantly increased the number of fungi (Figure 6B): the fungi of B1 and B2 were 80.38 and 159.85% higher than those of B0, respectively. The application of biochar and inorganic and organic fertilizers had similar effects on actinomycetes as eukaryotes. Both biochar and organic fertilizer could significantly increase the number of actinomycetes (Figure 6C). The actinomycetes of B1 and B2 were 56.45 and 111.59% higher than B0, respectively. The actinomycetes of B0F1M1 was 18.12% higher than B0F1M0; B1F1M1 was 22.34% higher than B1F1M0; and B2F1M1 was 11.34% higher than B2F1M0. The application of biochar with inorganic and organic fertilizers significantly affected the gram-negative bacteria and the gram-positive bacteria (Figures 6D,E). The low-rate application of biochar significantly increased the number of the gram-negative bacteria and the gram-positive bacteria: the gram-negative bacteria and the gram-positive bacteria of B1 increased by 114.42 and 50.91%, respectively, compared with B0. The low-rate application of biochar also significantly increased the number of protozoa (Figure 6F): the protozoa of B1 increased by 70.08% compared to B0.

### Linking PLFA and soil fertility properties to the combined application of biochar and organic and inorganic fertilizers

**Figure 7:**
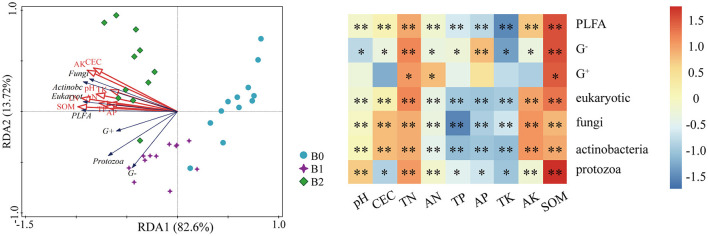
The redundancy analysis (RDA) and heatmap based on different treatments and microbial community compositions and soil fertility properties. * and ** Significant difference at *p* = 0.05 and *p* = 0.01, respectively.

Relationships of different treatments and microbial community compositions and soil fertility properties were illustrated by RDA analysis (Figure 7). Soil microbial community compositions accounted for 96.2% of the soil fertility. Specifically, the fungi, actinobacteria, eukaryote and PLFA were significant positively correlated with soil fertility. The protozoa, gram-negative and gram-positive bacteria were not strongly correlated with soil fertility. Moreover, the contributions of different application rates of biochar to soil fertility were also different, which was ordered as: B2>B1>B0.

## Discussions

### The effects of biochar combined with organic and inorganic fertilizers on rapeseed nutrients and oleic acid content

This study showed that the combined application of biochar, inorganic fertilizer and organic fertilizer could improve the absorption and utilization of nutrients in rapeseed. Widowati and Asnah (31) found that biochar application increased crop uptake of potassium by 128%. Xiang et al. (41) explained that biochar modified the soil environment thus promoting crop root growth and soil nutrient absorption. Nigussie et al. (40) found that biochar significantly increased nutrient absorption by crops. This study showed that the application of biochar could improve the absorption and utilization of nitrogen and potassium in rapeseed, but there was no significant improvement effect on the absorption and utilization of phosphorus. At the same time, the application of organic fertilizers and inorganic fertilizers could improve the absorption and utilization of nitrogen and phosphorus in rapeseed, and the application of organic fertilizers could significantly improve the absorption and utilization of potassium. Tan et al. (42) showed that the combined of biochar and organic fertilizer could significantly increase the nitrogen concentration of peanut grains. At the same time, the combined of biochar and organic and inorganic fertilizers could promote the nitrogen absorption of peanuts. And the research showed that soil pH, total organic carbon, available nutrients, the yield and qualities (reduced sugar, soluble protein, and soluble solid) of red pitaya increased with the application of organic fertilizer compared with no application of biochar and organic fertilizer, but the combined application of biochar and organic fertilizer was more effective than their sole application (43). Jaiswal et al. (44) showed that biochar had a priming effect on tomato growth-related genes expression. This study explained the promoting effect of biochar on plant nutrient absorption from the perspective of molecular biology.

Oleic acid is a fatty acid that is a healthier source of fat in the diet (28). This study found that the application of biochar had little effect on the oleic acid in rapeseed, but the application of organic fertilizer could significantly increase the oleic acid content in rapeseed, indicating that the application of biochar did not reduce the quality of rapeseed while promoting crop growth and development, and the combination of organic fertilizer could significantly improve the quality of rapeseed.

### The effects of biochar combined with organic and inorganic fertilizers on soil fertility and soil microbial communities

This study showed that the application of biochar in combination with inorganic and organic fertilizers could improve soil fertility. Application of biochar was able to significantly increase soil pH and CEC. This finding was consistent with Martinsen et al. (45) who explained that biochar application in the soil could react a liming effect. It was understood because biochar was alkaline in nature and dissociation of phenolic –OH groups in biochar increased its net negative surface charge (46). The presence of organic fertilizer increased soil pH, because plant absorbed more cations (positive charges) than anions (negative charges), and the combined application of inorganic and organic fertilizers were generally useful to sustain pH and soil fertility (47).

This study showed that the application of biochar could increase the nitrogen and potassium content in the soil and reduce the loss of nitrogen and potassium but had little effect on phosphorus. The application of organic fertilizer could increase the content of nitrogen, phosphorus and potassium, especially the content of soil available phosphorus. These findings were consistent with Widowati and Asnah (31), who found that biochar could act as a sorbent for both organic and inorganic fertilizers, thereby increasing crop yields and reducing fertilizer requirement. This was because biochar could attract and retain soil nutrients (11). Meanwhile, Jaiswal et al. (44) found that biochar application under low P input supported a more organized *phoD* gene community and preferentially enriched taxa in terms of their capacity for P mineralization, which in turn might enhance P bioavailability and plant P acquisition. It had been shown that biochar amendment could alter soil N cycling by altering microbial functional genes abundance and richness (48). However, for phosphorus, exchangeable aluminum in acidic soils would combine with phosphorus to form insoluble aluminum phosphorus, thereby reducing the availability of phosphorus. The purple soil was alkaline soil, so this might be the reason why the use of biochar had little effect on the phosphorus content (49, 50). Van Zwieten et al. (17) showed that the positive effects of biochar treatment on plant growth were due to its consequences on plant nutrition. This study showed that the application of biochar in combination with inorganic and organic fertilizers could significantly increase SOM. This was because biochar itself was an organic material with a high carbon content, and its application to the soil increased the organic matter in the soil (51). At the same time, the application of organic fertilizer also significantly increased the soil organic matter content. As soil conditioners with high organic matter content, biochar and organic fertilizer have a very significant impact on soil organic matter, and the impact of their own organic matter on soil even exceeds the synergistic effect of biochar, organic fertilizer and inorganic fertilizer.

Biome diversity was critical to soil function and had important implications for controlling nutrient cycling, increasing water use efficiency, enhancing soil aeration, and maintaining soil structure and stability (52). This study showed that biochar application could significantly increase the PLFA of the soil. With the application of biochar and organic fertilizer, the PLFA of the soil increased significantly. Li et al. (53) found that although the effects of biochar on soil microbial diversity were different, it was certain that biochar application had increased microbial biomass. This study found that eukaryotes, fungi and actinomycetes under high-rate biochar application were higher than those under low-rate biochar application; but for gram-negative bacteria, gram-positive bacteria and protozoa, they were higher under low-rate biochar than that under high-rate biochar. This was inconsistent with the study of Yuan et al. (54), possibly due to differences in soil type and the type of biochar applied. The study of Jin (49) showed that different types of biochar, when applied in different types of soil, had different effects on soil microbes. However, it was certain that the combination of biochar and organic and inorganic fertilizers could improve the richness of soil microbial communities. Lehmann et al. (13) elucidated that the efficacy of biochar on soil microorganism might be determined by both physical and chemical properties of biochar. We demonstrated that biochar could significantly increase soil pH, CEC, SOM and TOC, and could significantly increase the nitrogen, phosphorus and potassium content in soil and plants. This result implied that soil microbial community was encouraged by availability of nutrients in the soil. Gul et al. (55) explained that the presence of inorganic and organic fertilizer as co-amendment prevented nutrient deficiency for microbial growth. Moreover, DeLuca et al. (56) proved that in farming practice, biochar was frequently applied together with fertilizer, as biochar was not fertilizer, and it did not carry a lot of readily available nutrients. In brief, biochar application in soil could change soil physiochemical properties and mediate electron transfer processes, thereby improving metabolism of microbial community (15, 57, 58).

This study showed that soil fertility was related to soil microbial communities, being consistent with previous studies (59–61), in which soil fertility could affect soil microbial growth, and soil microbial richness could also affect soil fertility. Luo et al. (62) found that microbial communities were more active in high-yielding soils in a study on two types of dryland paddy soils. This study showed that there was a strong relation between the amount of biochar application and soil fertility. This study showed that soil fertility, especially SOM, had the highest contribution to microbial community diversity. Li et al. (63) showed that SOM was used to predict soil microbial activity. Stefanowicz et al. (64) believed that SOM had the strongest positive effect on most microbial parameters. This study supported the conclusion of previous studies, and this study also pointed out that K was a large contributor to the effects of fungi and actinobacteria, while gram-negative bacteria and gram-positive bacteria were weakly correlated with soil fertility.

## Conclusion

The application of biochar in combination with organic and inorganic fertilizers could promote the nutrient uptake of rapeseed and improve the soil fertility and microbial environment. Low-rate of biochar could promote rapeseed nutrient uptake, improve soil fertility, and increase soil PLFA. However, excessive biochar application was not conducive to sustainable farming. Therefore, we recommend the application of low-rate biochar (35 t/ha) in combination with organic fertilizer (4.5 t/ha) and low-rate inorganic fertilizers (30 kg/ha N, 87.5 kg/ha P_2_O_5_ and 60 kg/ha K_2_O) in rapeseed production in purple soil area of southwest China.

## Data availability statement

The original contributions presented in the study are included in the article/Supplementary material, further inquiries can be directed to the corresponding author/s.

## Author contributions

LW, CL, SM, and ML contributed to conception and design of the study. CL and SM organized the database and performed the statistical analysis. ML wrote the manuscript. QM, WS, LS, KC, MS, and YZ participated in the experiment and collected data. All authors contributed to the article and approved the submitted version.

## Funding

This work was supported by Natural Science Foundation Project of China (No. 31871583) and Special Fund for Agro-Scientific Research in the Public Interest (No. 201503127).

## Conflict of interest

The authors declare that the research was conducted in the absence of any commercial or financial relationships that could be construed as a potential conflict of interest.

## Publisher's note

All claims expressed in this article are solely those of the authors and do not necessarily represent those of their affiliated organizations, or those of the publisher, the editors and the reviewers. Any product that may be evaluated in this article, or claim that may be made by its manufacturer, is not guaranteed or endorsed by the publisher.
